# Three-Generation Study of Male Rats Gestationally Exposed to High Butterfat and Bisphenol A: Impaired Spermatogenesis, Penetrance with Reduced Severity

**DOI:** 10.3390/nu13103636

**Published:** 2021-10-17

**Authors:** Shuk-Mei Ho, Rahul Rao, Bin Ouyang, Neville N. C. Tam, Emma Schoch, Dan Song, Jun Ying, Yuet-Kin Leung, Vinothini Govindarajah, Pheruza Tarapore

**Affiliations:** 1 Department of Pharmacology and Toxicology, University of Arkansas for Medical Sciences, Little Rock, AR 72205, USA; nevilletam@uams.edu (N.N.C.T.); rickyleung@uams.edu (Y.-K.L.); 2 Central Arkansas Veterans Healthcare System, Little Rock, AR 72205, USA; 3 Department of Environmental and Public Health Sciences, University of Cincinnati Medical Center, Cincinnati, OH 45267, USA; raorl@ucmail.uc.edu (R.R.); ouyangb@ucmail.uc.edu (B.O.); berryec@mail.uc.edu (E.S.); songd@ucmail.uc.edu (D.S.); jying@uams.edu (J.Y.); 4 Center for Environmental Genetics, University of Cincinnati Medical Center, Cincinnati, OH 45267, USA; 5 Department of Biostatistics, University of Arkansas for Medical Sciences, Little Rock, AR 72205, USA; 6 Stem Cell Program, Division of Experimental Hematology and Cancer Biology, Cincinnati Children’s Hospital Medical Center, Cincinnati, OH 45229, USA; vinothini.janakiram@cchmc.org; 7 Cincinnati Cancer Center, Cincinnati, OH 45267, USA

**Keywords:** testes, endocrine disrupting chemicals, high-fat butter, bisphenol A, aromatase, MBD3, ERbeta

## Abstract

Gestational high butterfat (HFB) and/or endocrine disruptor exposure was previously found to disrupt spermatogenesis in adulthood. This study addresses the data gap in our knowledge regarding transgenerational transmission of the disruptive interaction between a high-fat diet and endocrine disruptor bisphenol A (BPA). F0 generation Sprague-Dawley rats were fed diets containing butterfat (10 kcal%) and high in butterfat (39 kcal%, HFB) with or without BPA (25 µg/kg body weight/day) during mating and pregnancy. Gestationally exposed F1-generation offspring from different litters were mated to produce F2 offspring, and similarly, F2-generation animals produced F3-generation offspring. One group of F3 male offspring was administered either testosterone plus estradiol-17β (T + E2) or sham via capsule implants from postnatal days 70 to 210. Another group was naturally aged to 18 months. Combination diets of HFB + BPA in F0 dams, but not single exposure to either, disrupted spermatogenesis in F3-generation adult males in both the T + E2-implanted group and the naturally aged group. CYP19A1 localization to the acrosome and estrogen receptor beta (ERbeta) localization to the nucleus were associated with impaired spermatogenesis. Finally, expression of methyl-CpG-binding domain-3 (MBD3) was consistently decreased in the HFB and HFB + BPA exposed F1 and F3 testes, suggesting an epigenetic component to this inheritance. However, the severe atrophy within testes present in F1 males was absent in F3 males. In conclusion, the HFB + BPA group demonstrated transgenerational inheritance of the impaired spermatogenesis phenotype, but severity was reduced in the F3 generation.

## 1. Introduction

Barker’s hypothesis of developmental origin of health and disease (DOHaD) is now well documented [1,2,3,4]. Maternal nutrition, such as food restriction or excessive fat intake during pregnancy and/or lactation, significantly impacts the health of offspring [1,5]. Similarly, maternal exposure to a wide variety of environmental toxicants, including endocrine-disrupting chemicals (EDCs), during pregnancy and/or lactation changes the disease risk profiles of the offspring [6,7,8,9,10]. Ample evidence now indicates that in utero or early-life exposure to specific nutritional, chemical, or lifestyle stressors can increase disease risk later in life through epigenetic reprogramming of gene expression in target organs [11,12,13,14]. One limitation of such studies is their focus. Most studies have been centered on the effects of a single stressor, e.g., a specific environmental toxicant or type of diet. Few have studied the consequences of exposure to a multitude of stressors and their interactive effects [15]. Even rarer are studies that seek to determine if such interactions are inheritable. Because human exposure is almost always multifactorial, any synergism or antagonism among stressors and their likelihood to be inherited will greatly increase the complexity of how altered traits are passed down to successive generations. Thus far, no studies have addressed the transgenerational transmission of disruptive interactions between a high-fat diet and endocrine disruptors. To fill this data void, we designed this study to determine if early-life (gestation) exposure to a high-butterfat diet (HFB) and bisphenol A (BPA), alone or in combination, have transgenerational effects on spermatogenesis. 

Bisphenol A (BPA) is a ubiquitous EDC. According to the World Health Organization [16], an endocrine disruptor is an exogenous substance or mixture that alters hormone functions and consequently causes adverse health effects in the exposed human or animal and their progeny and in subsequent generations. Endocrine disruptors such as BPA are associated with human diseases such as cardiovascular disease, diabetes, obesity, immunotoxicity, and infertility, and with adverse mental and reproductive health outcomes [17,18,19,20]. BPA is ubiquitously present in the environment [21] and is found in 93% of the population, as detected in a large-scale cross-sectional US study involving 2,517 participants of the 2003–2004 National Health and Nutrition Examination Survey (NHANES) [22]. BPA was found in the serum, urine, placenta, breast milk, and umbilical cord serum of a birth panel of mother–neonate pairs [23,24], thus supporting the occurrence of in utero exposure to these chemicals. Pregnancy is a window for susceptibility to environmental toxicants, and exposure to BPA during pregnancy has been linked to adverse outcomes at birth and later in life [15,25,26,27,28,29,30,31,32]. In particular, we found that embryonic exposure to BPA (and HFB) influences spermatogenesis in the exposed F1-generation offspring as they become middle aged [15,33], and also under conditions that mimic aging, such as exposure to testosterone plus estradiol-17β (T + E2). 

Animal studies have indicated that foods high in fat content, regularly found in the Western diet, are potential disruptors of male and female reproductive capacity [34,35,36,37]. Combined parental obesity has been found to detrimentally impact preimplantation mouse embryo development, kinetics, morphology, and metabolism [38]. Today, greater than 38% of adults around the world are defined as overweight (BMI ≥ 25 kg/m^2^) and 13% are defined as obese (BMI ≥ 30 kg/m^2^) [39]. This is a cause for increasing concern regarding the influence of maternal and paternal diets before and during pregnancy on the reproductive capacity of their offspring and across multiple generations [40,41]. 

Fetal exposure to EDCs and high-fat diets [35,42,43] can cause abnormalities in the F1 and F2 generations. Both the F1-generation embryo and F2-generation germ line are directly exposed when an F0-generation pregnant mother is exposed. Therefore, the F3 generation is the first unambiguous transgenerational generation that should be studied for possible transmission effects of exposure. Essentially, there is now increasing evidence of F2- and F3-generation transference of the effects of diet or endocrine disruption [30,44,45,46,47,48,49,50,51].

We previously reported that maternal consumption of HFB exacerbated the adverse effects of BPA on mammary cancer risk [26] and spermatogenesis [15,33] in the offspring. Similarly, others have reported that a maternal high-fat diet worsened early-life BPA-induced male hypertension [52] and glucose metabolism disorder [53] in adult offspring. Yet when studying the periadolescent social play of specific offspring, no interactive effects between a perinatal high-fat diet and BPA were observed. In real human scenarios, environmental EDC exposure is often under different dietary conditions. In the US, a high-fat Western diet with >35% saturated fat is common. Therefore, a more meaningful way to study the developmental impact of early-life EDC exposure is to query the synergistic/antagonistic effects of a high-fat diet during gestation and/or lactation. 

In this report, our objective was to determine whether the impaired spermatogenesis phenotype observed in middle-aged adult F1 male rat offspring exposed to HFB and/or BPA in utero could be transmitted to the F3 males. We examined spermatogenesis in the testes of F3 rats using the hormone treatment rat model and in 18-month-old rats (equivalent to 45 human years [54]). We found that the rats gestationally exposed to HFB and BPA exhibited impaired spermatogenesis as they became middle aged (as per the T + E2 model), but with reduced severity than the middle-aged F1-generation rats. Moreover, while 50% of the 18-month-old rats in the F3 generation HFB + BPA group shows impaired spermatogenesis, significance was not observed between groups.

## 2. Materials and Methods

### 2.1. Animals and Diets

The animal usage and care protocols were approved by the Institutional Animal Care and Use Committee at the University of Cincinnati and were in compliance with NIH guidelines. As outlined in our previous publication [15,26], female Sprague-Dawley (SD) rat dams were housed under BPA-free conditions [15,33], and randomly placed into groups according to the in utero diet exposure for male offspring: (1) the American Institute of Nutrition (AIN) group was exposed to a modified open standard diet (product #D04042310 AIN 93G (Research Diets, Inc., New Brunswick, NJ, USA), 10 kcal% butterfat) certified to contain no phytoestrogens; (2) the BPA group was exposed to the test compound BPA at 25 µg/kg body weight per day (kg bw-d), which was directly incorporated into the unsupplemented (AIN) pellet diet; (3) the HFB group was exposed to 39 kcal% butterfat, which was directly incorporated into the unsupplemented (AIN) pellet diet; and (4) the HFB + BPA group was exposed to 25 µg/kg bw-d BPA and 39 kcal% butterfat. BPA-free conditions imply special cages and water sources/bottles that are certified BPA-free, and a separate room with filters for this study. 

Male pups were transferred to the normal (non-BPA-free) environment at PND 21 and only transferred back to the BPA-free environment during mating. All female offspring used for mating were maintained in a BPA-free environment throughout the study. The number of animals in each diet group was counted by litter (i.e., one male offspring per litter). F1-generation offspring from different litters were mated within each group to produce F2 offspring, and similarly, F2-generation animals produced F3-generation offspring. In brief, both F1-generation progenitors (males and females) were gestationally exposed to BPA and/or HFB, but only the F0 generation was fed the respective diets. Sibling crossing was avoided during mating. In other words, only the founder F0 pregnant rats were fed the experimental diets, thus the male and female F1 offspring were only exposed to these diets in utero. At birth and thereafter, they were maintained on the AIN diet, as were subsequent generations. It should be noted that the F0 breeder males were briefly exposed to the experimental diets during the mating period.

### 2.2. Hormone Treatment SD Rat Model

At least 11 male pups per group, 2 F3 male pups per litter, were used in the hormone treatment SD rat model, as detailed for the F1-generation animals [15,26], to mimic aging. At 10 weeks of age, one male offspring per litter was surgically implanted with silastic capsules packed with T + E2 (MilliporeSigma, St. Louis, MO, USA) as described previously [15,55] or with an empty capsule. Briefly, the hormone consists of a 2 cm long capsule (2 pieces) containing T (MilliporeSigma) and one 1 cm long capsule packed with E2 (MilliporeSigma). These capsule lengths result in serum concentrations of ~75 pg/mL E and 3 ng/mL T [56]. At 30 weeks of age, rats were sacrificed. One testis was fixed in formalin and embedded in paraffin, and the second testis was frozen in Tissue-Tek O.C.T. compound (Sakura Finetek USA, Torrance, CA, USA). While we started with 15 pups per group, the differences in litter numbers in the various treatment groups reflect that some pups were sacrificed due to difficult postsurgical healing, or data were eliminated due to leakage of capsules. 

### 2.3. Natural Aging Model

One male F3 pup from each litter was used in the aging study (7 pups for the AIN group, 5 for the BPA25 and HFB groups, and 6 for the HFB + BPA group) to examine the testes when rats are “middle-aged”. Briefly, these male rats were fed an AIN diet and the adult rats were sacrificed at 18 months (PND 540), when the endogenous E2:T ratio was previously shown to be highest [57]. The testes were collected as mentioned above. While we started with 7 pups per group, the differences in experimental numbers for the various treatment groups reflect that during the 18 months, some rats had to be sacrificed due to lethargy or health distress, or they died before PND 540 due to unknown causes. 

### 2.4. Tissue Collection and Immunohistochemistry

Immunohistochemistry (IHC) analyses were performed as previously described [15]. Testis sections were deparaffinized and antigen retrieval was performed. Following antigen retrieval, the sections were pre-incubated with 5% normal goat serum in PBS. Sections were then incubated with CYP19A1 (Santa Cruz Biotechnology, Inc., Santa Cruz, CA, USA) or estrogen receptor 2 (ESR2)/ERbeta (BioGeneX, Fremont, CA, USA) antibodies (Table 1) diluted in blocking solution and visualized using the avidin–biotin–peroxidase complex with biotinylated anti-rabbit secondary antibodies (Vector Laboratories, Inc., Burlingame, CA, USA). Samples were processed with the Vectastain Elite ABC kit (Vector) and DAB chromogen (Sigma, St. Louis, MO, USA), and 100 to 400 seminiferous tubules (STs) were scored per rat. All slides were read in a blinded fashion, and data were analyzed.

On the CYP19A1 stained sections, 100 to 120 STs were scored for the last step of differentiated germ cells (spermatogenesis) present within the tubules. We assessed the incidence of tubule atrophy (spermatogonia and Sertoli cell-only tubules) in adult male rats from each treatment group by visual analysis of hematoxylin and eosin stained sections for each animal. We counted the number of tubules showing the presence and absence of ERbeta in cytoplasm and nucleus of round spermatids; the results represent the percentage of tubules. Similarly, we counted the number of tubules showing the presence and absence of CYP19A1 in the acrosome of spermatid stages 1–8, 9–12, and 13–16; the results represent the percentage of tubules. 

### 2.5. Quantitative Real-Time PCR (qPCR)

Total RNA was isolated from frozen testes using TRIzol reagent (Thermo Fisher Scientific, Waltham, MA, USA) and reverse transcribed using the Superscript IV One Step RT-PCR kit (Thermo Fisher Scientific) according to the manufacturer’s instructions. Quantitative PCR reactions were performed using PerfeCTa SYBR Green FastMix, Low ROX (Quantabio, Beverly, MA, USA), and monitored with the ViiA 7 Real-time PCR Detection System (Thermo Fisher Scientific). The primer sequences are presented in Table 2; some of these sequences have been previously published [58]. Individual mRNA levels were normalized to ribosomal protein L19 (*Rpl19*) and expressed relative to AIN control RNA levels. All data groups were analyzed by one-way ANOVA followed by Tukey’s post hoc or Dunnett’s multiple comparison test using Prism software (GraphPad, San Diego, CA, USA).

### 2.6. Statistical Analysis

**Figure 1:**
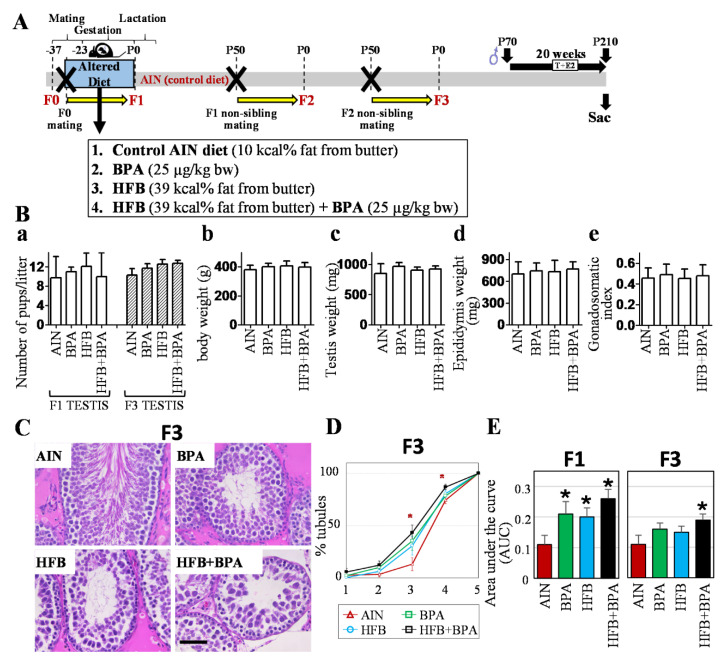
Impaired spermatogenesis in testes of T + E2−implanted F3-generation male offspring. (**A**) Scheme of dietary exposure groups, mating and T + E2 treatments. F0 dams were fed indicated diets during mating and gestation. Diets were changed to AIN diet after pups were born. Pups and their descendants were fed AIN diet. On PND 50, gestationally exposed F1 males and females from different litters were mated within each group, resulting in F2 offspring. Again, on PND 50, F2 offspring were similarly mated, resulting in F3 offspring. F3 males underwent T + E2 implantation. (**B**) Litter size and weight of male offspring (T + E2 model): (**a**) litter size for F1 and F3 offspring, body weight (bw) and weight of reproductive organs of male offspring for T + E2−exposed offspring (PND 210), (**b**) body weight, (**c**) right testis, (**d**) right epididymis, and (**e**) gonadosomatic index (weight of both testis/bw × 100). Error bars indicate standard deviation. No significance was found using one-way ANOVA. (**C**) Representative tubules illustrating predominant phenotype observed for each diet group. T + E2−implanted F3 offspring from control AIN diet group show all stages of spermatogenesis culminating in spermatozoa. BPA, HFB, and HFB + BPA group offspring show impaired spermatogenesis, with last step predominantly consisting of round and/or condensing spermatids. Bar = 60 µm. (**D**) Plot of tubules showing cumulative progression of spermatogenesis. STs were scored for spermatogenesis, showing (1) spermatogonia/atrophic tubules, (2) spermatocytes, (3) round step 8 spermatids, (4) condensed step 16 spermatids, and (5) spermatozoa as last differentiated germ cell present within STs. Curves represent progression of spermatogenesis per subject. (* *p* < 0.05 vs AIN group). (**E**) Comparison of area under the curve (AUC) as a measure of spermatogenesis among groups. (* *p* < 0.05). Two sections examined per animal.

For Table 3 and Table 4, Fisher’s exact test was used to calculate the odds ratio using Prism software. Similar to our previous publication [15], a spermatogenesis curve was plotted from the scores summed up to specific spermatogenesis steps as cumulative values showing (1) spermatogonia/atrophic tubules, (2) spermatocytes, (3) round step 8 spermatids, (4) condensed step 16 spermatids, and (5) spermatozoa (Figure 1B). The area under the curve (AUC) was considered as a measure of overall progression of spermatogenesis (Figure 1C). The AUC ranged from 0 to 1, with higher values indicating more severe impairment of spermatogenesis.

The numerical measure of overall spermatogenesis was assessed for its association with the fixed effects of diet (AIN vs. HFB and BPA vs. none) and their interaction using a fixed effect (or two-way ANOVA) model. Post hoc means were estimated from the model and compared among groups. Considering that a small sample size was used in each group in this study, such comparisons did not account for any multiple comparison method in the analysis. In order to ensure that the results were robust and invariant to the statistical methods, other competing statistical models, the non-parametric Wilcoxon rank sum test and a cumulative logistical regression model (after using quintiles of the original measure), were used. Because all methods showed similar findings from the analyses, only the results from the primary method of the fixed effect model are presented in this paper. All statistical analyses were computed using SAS 9.4 software (SAS Institute, Cary, NC, USA). *p*-values < 0.05 were considered statistically significant. Pearson correlation coefficients were calculated using two-tailed *p*-values with all diet groups.

## 3. Results

### 3.1. Prenatal HFB + BPA Exposure Induces Significant Spermatogenesis Arrest in T + E2 Implanted Offspring 

Male F1 offspring were exposed in utero to AIN, BPA, HFB, and HFB + BPA. At birth, they were fed an AIN diet. We had shown that adult F1 males with gestational exposure to BPA, HFB, or HFB + BPA, in both the naturally aged model and the T + E2-implanted model (a hormonal milieu previously reported to mimic aging [57,59]), exhibited impaired spermatogenesis [15], while the sham-implanted gestational exposed groups had normal spermatogenesis. F1-generation offspring from different litters were mated within each group to produce F2 offspring, and similarly, F2-generation animals produced F3-generation offspring (Figure 1A). The scheme for the F1-generation T+E2 treatments was similar, and was outlined in our previous publication [15,33]). Similar to our results with the F1-generation mating (Figure 1(Ba)), we did not observe any significant litter size differences between the various groups of F2-generation mating (which resulted in F3-generation offspring). These F3 offspring underwent T + E2 implantation for 20 weeks (see Materials and Methods). No significant difference was observed in the body weight of F3-generation male offspring in the AIN, BPA, HFB, and HFB + BPA groups (Figure 1(Bb)). Also, no statistical significance was observed in testes (Figure 1(Bc)), epididymis weight (Figure 1(Bd)), or gonadosomatic index (Figure 1(Be)) between the groups. Also, no statistical difference was observed between the weights of F3-generation newborn male pups between these groups (results not shown). Figure 1C shows representative tubules illustrating the predominant phenotype observed for each diet group. We found that 91% of the AIN group offspring (Table 3) showed normal spermatogenesis (presence of spermatozoa in ≥14% of STs). The number of animals with normal spermatogenesis decreased in the BPA group offspring (71%) and the HFB group offspring (64%), but was not significantly different from the AIN group. On the other hand, only 40% of the HFB + BPA group had normal spermatogenesis in the STs, which was significantly different from the AIN group (*p* = 0.014). 

We next compared overall impaired spermatogenesis among groups. For this, we plotted a curve reflecting the extent of spermatogenesis within the tubules for each diet (Figure 1D). When the curves for F3 offspring were compared, it was evident that the AIN curve showed a slight increase at the round spermatid step, but the majority of the increase was at the condensed spermatid step. On the contrary, the average curves of the BPA, HFB, and HFB + BPA groups showed higher slopes for the round spermatid stage, indicating a disruption in spermatogenesis at an earlier stage in the F3 groups, consistent with that observed for the F1 offspring. However, among the F1 offspring, 50% of the animals in the HFB + BPA group exhibited a high percentage of atrophic tubules [15], compared to only 13% for the F3-generation litter. This dramatic decrease in atrophic tubules in the HFB + BPA group between generations indicates that the severity of the impaired tubule phenotype declined in the F3 litters. Additionally, in F1 offspring, the means of overall impaired spermatogenesis (or AUC) in the BPA, HFB, and HFB + BPA groups were significantly higher than those of the AIN groups (Figure 1E, left panel). For the F3 offspring, only the AUC of the HFB + BPA group was significantly higher than that of the AIN group (Figure 1E, right panel). 

**Figure 2:**
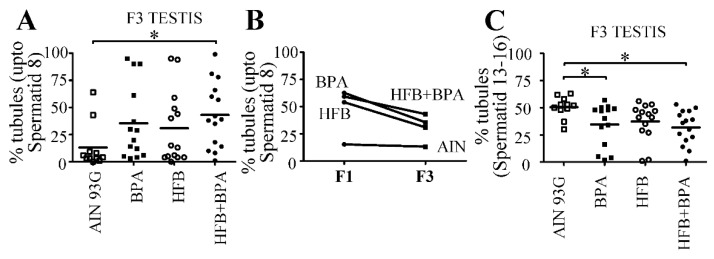
Semi-quantification of impaired spermatogenesis. Numbers of (**A**,**B**) STs with spermatogenesis up to round spermatid stage and (**C**) 13–16 spermatid stage were tallied for T + E2−implanted F1 and F3 male offspring in diet groups as indicated. * *p* < 0.05 by one-way ANOVA between groups indicated, followed by Dunnett’s multiple comparison test. Each symbol represents one animal per litter. Two sections examined per animal.

Finally, for the T + E2-implanted F3-generation offspring, we found that the BPA, HFB, and HFB + BPA groups exhibited higher percentages of STs with round spermatid cells as the final step in spermiogenesis (Figure 2A) compared to the AIN group. However, the BPA and HFB groups showed a trend (not significant) of impaired spermatogenesis, while the HFB + BPA group showed a significant disruptive effect (*p* < 0.05) compared to the AIN group. When comparing each group across the F1 and F3 generations, we found that compared to each F1 group, the corresponding F3 group did not show a significant change in the average number of impaired STs (Figure 2B), arguing for a transgenerational effect of disrupted spermatogenesis for the BPA, HFB, and HFB + BPA groups. 

### 3.2. CYP19A1/Aromatase Expression during Spermatogenesis in T + E2-Implanted Offspring

**Figure 3:**
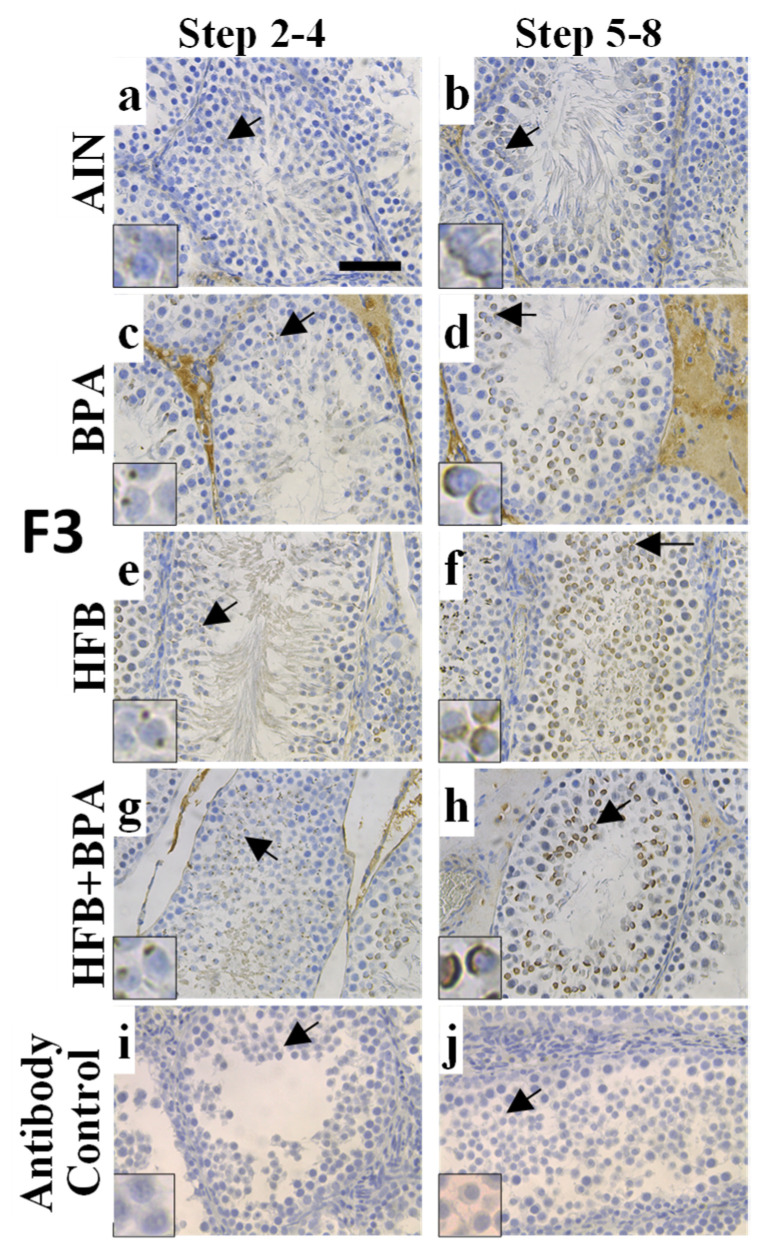
Cyp19A1 staining localizes to acrosomes in T + E2−treated offspring. Representative tubules illustrating acrosomal staining within (**a**,**c**,**e**,**g**,**i**) spermatid steps 2–4 and (**b**,**d**,**f**,**h**,**j**) spermatid steps 5–8 using anti-Cyp19A1 antibody. (**i**,**j**) Negative control (no primary antibody). Bar = 60 µm.

**Figure 4:**
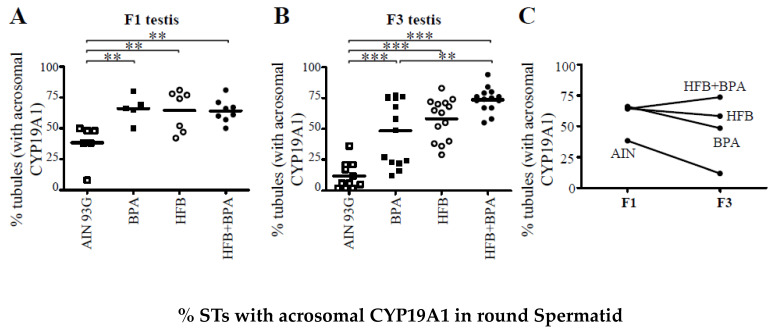
Increased expression of acrosomal CYP19A1 in round spermatids of F1 and F3 T + E2−treated HFB and HFB + BPA groups. (**A**) F1 and (**B**) F3 testes were scored for number of STs with acrosomal CYP19A1 in round spermatids. Each symbol represents a pup from an independent litter. (**C**) Comparison of F1 and F3 CYP19A1 expressing tubules. ** *p* < 0.01, *** *p* < 0.001 using one-way ANOVA between groups indicated, followed by Dunnett’s multiple comparison test.

In our initial analysis [15], we quantitated the overall intensity of CYP19A1 expression in all spermatids and found no significant changes in expression between the groups. For this study, we examined the intensity and localization of CYP19A1 at round spermatid step 1, steps 2–4, and steps 5–8. We found an interesting pattern emerge. IHC staining showed an absence of CYP19A1 in spermatid step 1. However, CYP19A1 expression was observed on the expanding acrosome of round spermatid steps 2–4 and 5–8 (Figure 3). Also, while the intensity and acrosomal presence of CYP19A1 was low in round spermatids of the AIN group, the larger number of round spermatids of the BPA and HFB groups, and especially the HFB + BPA group, showed the strong acrosomal intensity of CYP19A1 staining (Figure 3). Next, the testes were scored for the number of STs with acrosomal CYP19A1 staining in round spermatids. When examining the round spermatids of F1- and F3-generation testes, a significant number of animals had an increased number of tubules with acrosomal CYP19A1 in the BPA, HFB, and HFB + BPA groups compared to the respective AIN groups (Figure 4A,B). It should be noted that the AIN group showed a significantly lower number of STs with acrosomal CYP19A1 localization in the F3-generation testes compared to the F1 generation. Hence, we also compared the F1 AIN group to the F3 diet groups by one-way ANOVA. Only the F3 HFB + BPA group showed significant differences in the number of STs with acrosomal CYP19A1 localization compared to the F1 AIN group (Figure 4A,B). Also, when comparing groups in the F1 and F3 generations, there was no significant change in the number of tubules with acrosomal CYP19A1 localization within the AIN, BPA, HFB, and HFB + BPA groups (Figure 4C), arguing for transgenerational inheritance of this phenotype.

### 3.3. Decrease in Cytoplasmic ERbeta Expression in Round Spermatids of T + E2-Implanted Offspring

**Figure 5:**
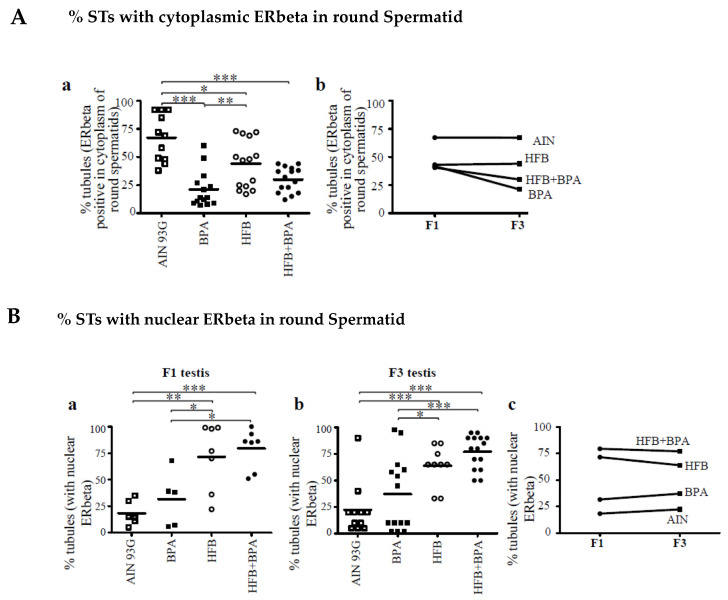
Cytoplasmic and nuclear expression of ERbeta in round spermatids (T + E2−treated). (**A**) Testis of F3-generation offspring were scored for number of STs with ERbeta staining in cytoplasm of round spermatids. Each symbol represents a pup from an independent litter. (**B**) Comparison of STs with ERbeta staining in nucleus of round spermatids within each group (AIN, BPA, HFB, and HFB + BPA) in (**a**) F1 and (**b**) F3 generation offspring. (**c**) Comparison of F1 and F3 nuclear ERbeta expressing tubules. * *p* < 0.05, ** *p* < 0.01, *** *p* < 0.001 using one-way ANOVA between groups indicated, followed by Dunnett’s multiple comparison test. Two sections examined per animal.

In our initial report [15], we showed a decrease or loss of expression of cytoplasmic ERbeta in the round spermatids of the T + E2-treated BPA, HFB, and HFB + BPA groups compared to the AIN group. We examined whether this phenotype persists in the F3 groups. IHC stained testes of the F3 T + E2-treated rats revealed a decrease or loss of expression of ERbeta in the cytoplasm of round spermatids in a significant number of animals in the BPA, HFB, and HFB + BPA groups (Figure 5(Aa)) compared to the AIN group. However, when comparing groups in the F1 and F3 generations, there was no significant change in cytoplasmic localization of ERbeta within the AIN, BPA, HFB, and HFB + BPA groups (Figure 5(Ab)), thus arguing for transgenerational inheritance of this phenotype.

### 3.4. Nuclear ERbeta Localization in Round Spermatids in T + E2-Implanted Offspring

Estrogen binds to its receptors ERalpha and ERbeta to translocate into the nucleus and exert cellular effects through the transcription of downstream targets [60]. We examined and scored the testes for the number of STs with nuclear ERbeta staining in round spermatids (Figure 5B). When examining the round spermatids of F1- and F3-generation offspring, we found an increased number of tubules with nuclear ERbeta in a significant number of animals in the HFB and HFB + BPA groups compared to the respective AIN groups (Figure 5(Ba,b)). We compared the F1 and F3 diet groups by one-way ANOVA. There was no significant change in nuclear ERbeta localization within the BPA, HFB, and HFB + BPA groups (Figure 5(Bc)) across generational offspring, again arguing for transgenerational inheritance of this phenotype.

### 3.5. Correlation between Acrosomal CYP19A1 and Nuclear ERbeta Localization in Round Spermatids

**Figure 6:**
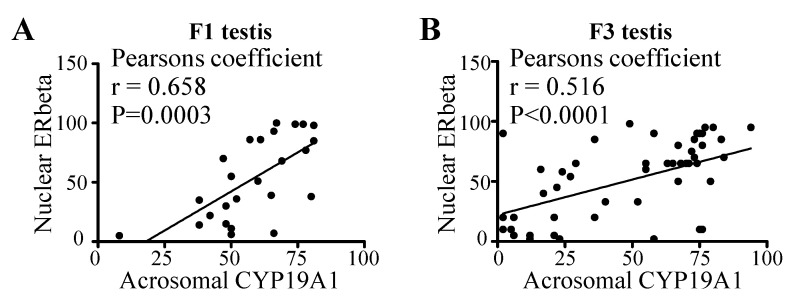
Correlation analysis of nuclear ERbeta and acrosomal CYP19A1 localization using pooled samples of all groups in the (**A**) F1 and (**B**) F3 generation testes. Each symbol represents a pup from an independent litter.

Exogenous estradiol has been shown to induce spermatogenetic disorders by influencing apoptosis and the estrogen receptor signaling pathway [61]. During perinatal life, endogenous estrogens are believed to mediate inhibition of male germ cell line development in mice using the ERbeta signaling pathway [62]. Because CYP19A1, an aromatase, mediates testosterone-to-estrogen conversion, we sought to determine whether a correlation exists between the acrosomal CYP19A1 and nuclear ERbeta localization. Indeed, we found a significant correlation between acrosomal CYP19A1 and nuclear ERbeta in both F1 (Figure 6A) and F3 (Figure 6B) generation animals (F1: Pearson r = 0.66, *p* = 0.0003; F3: Pearson r = 0.51, *p* < 0.0001).

### 3.6. 18-Month-Old F3-Generation Testes Exhibited Decreased Spermatozoa Numbers

**Figure 7:**
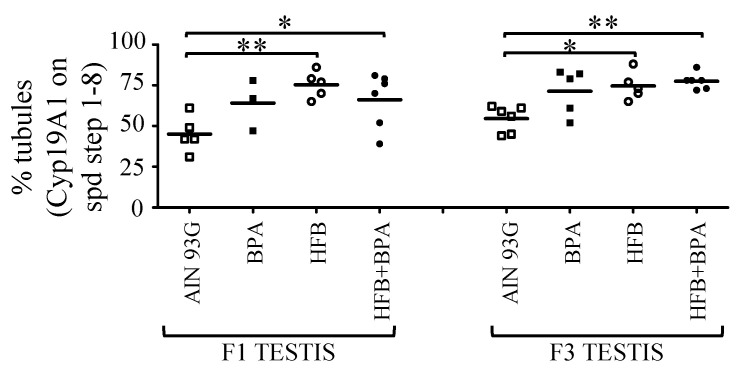
Increased expression of acrosomal CYP19A1 in round spermatids of aged animals, from F1- and F3-generation BPA, HFB, and HFB + BPA groups compared to AIN group. Testis sections were scored for number of STs with acrosomal CYP19A1 in round spermatids. Each symbol represents a pup from an independent litter. * *p* < 0.05, ** *p* < 0.01, using one-way ANOVA between groups indicated, followed by Dunnett’s multiple comparison test.

One male offspring per litter from the AIN, BPA, HFB, and HFB + BPA groups without any secondary exposure was sacrificed at approximately 18 months of age (~45 human years, or middle age [54]). It was shown previously that 18-month-old F1 offspring from the HFB + BPA group were 40 times more likely (statistically significant) to show abnormal spermatogenesis than those in the AIN group [15]. For the F3-generation litters, we found that while 86% of F3 offspring (18 months old) from the AIN group showed normal spermatogenesis (presence of spermatozoa in >20% of STs), only 50% (3 of 6 rats) of offspring from the HFB + BPA group had normal spermatogenesis (Table 4). The odds ratio suggests that abnormal spermatogenesis is 6 times more likely to occur in the HFB + BPA group versus the AIN group. However, spermatogenesis in the BPA, HFB, and HFB + BPA groups was not statistically significantly different from the control AIN group. Next, we examined the CYP19A1 expression pattern in older animals using IHC. A significant number of F1 and F3 offspring in the HFB and HFB + BPA groups showed CYP19A1 localization at the acrosome compared to the AIN group (Figure 7).

### 3.7. Methyl-CpG-Binding Domain (MBD3) Levels Are Reduced in HFB and HFB + BPA Group Offspring (T + E2-Treated)

**Figure 8:**
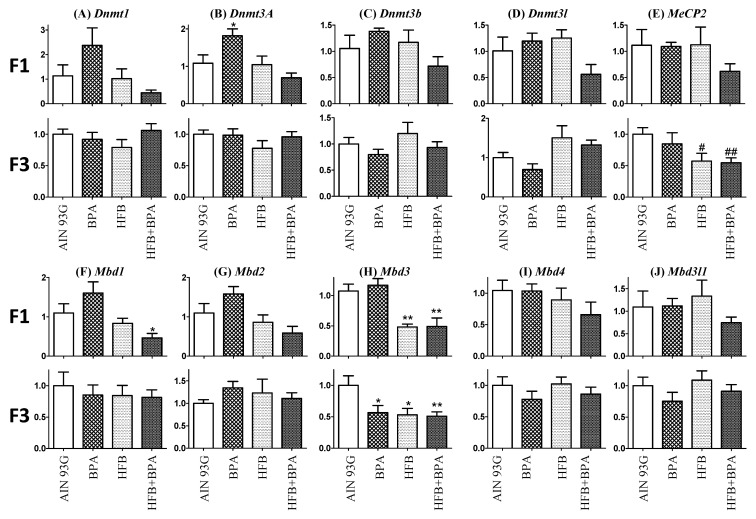
Relative gene expression was determined by real-time PCR for (**A**) *Dnmt1*, (**B**) *Dnmt3a*, (**C**) *Dnmt3b*, (**D**) *Dnmt3l*, (**E**) *MeCP2*, (**F**) *Mbd1*, (**G**) *Mbd2*, (**H**) *Mbd3*, (**I**) *Mbd4*, and (**J**) *Mbd3l1*. RNA was isolated from testes of F1- and F3-generation AIN, BPA, HFB, and HFB + BPA groups following T + E2 implantation. Relative expression ratio (RER) was calculated by normalizing transcript levels of a gene to that of *Rpl19* transcript level in the same sample. Levels of transcripts in all other treatment groups at various life stages were normalized to AIN controls. Statistical significant differences are indicated (* *p* < 0.05, ** *p* < 0.01 when comparing values with one-way ANOVA and # *p* < 0.05, ## *p* < 0.01 when compared by *T*-test with AIN group).

Previous studies found that overexpression of human-derived methyltransferase, hDNMT3A, in the testes of transgenic rats can induce genome-wide alterations in the DNA methylation pattern of rat sperm [63]. We studied the expression of DNA methyltransferases that are responsible for the formation of 5-methyl cytosine (DNMT1, DNMT3A, DNMT3B, DNMT3L) and methyl-CpG-binding domain proteins (MeCP2, MBD1, MBD2, MBD3, MBD3L1, MBD4). We found that for the F1 offspring, there was a trend toward decreased expression of mRNA for *Dnmt1*, *Dnmt3a*, *Dnmt3b*, *Dnmt3l*, and *MeCP2* in the HFB + BPA group compared to the AIN group. However, these changes were lost in the F3 offspring (except for MeCP2). Similarly, a trend was found in F1 offspring for decreased expression of mRNA for *Mbd1*, *Mbd2*, *Mbd3*, *Mbd3l1*, and *Mbd4*. Of special interest was the low expression of MBD3 that was maintained in the F3-generation offspring groups (Figure 8).

## 4. Discussion

The goal of this study was to evaluate whether the effects of BPA and HFB on male reproductive function are transferred across generations. Using a hormone treatment model, we found that the F3-generation offspring from the combination HFB + BPA diet groups had significantly disrupted spermatogenesis when compared to the AIN, BPA, and HFB groups. While the BPA and HFB groups showed a trend toward disrupted spermatogenesis, the effects were not as severe as those found in the respective F1-generation groups [15], and they lacked significance. Our data suggest that for the F3-generation offspring, HFB + BPA was detrimental to spermatogenesis. Furthermore, the detrimental effects on spermatogenesis in the HFB + BPA group occurred most significantly at the round spermatid stage. This is in contrast to the F1 offspring, in which the disruption in the HFB + BPA group started to occur at the spermatogonia–spermatocyte stage [15]. Finally, we observed a dramatic decrease in atrophic tubules in the F3-generation HFB + BPA group compared to the respective F1 group [15], further supporting that the severity of the penetrance effects of the exposure diets declined in the F3 litters. Notably, while the F1 pups were directly exposed to HFB and BPA (gestational exposure), the following generations were maintained on AIN diet only. This is reflected, for example, in the expression profile of genes involved in DNA methylation. While the BPA group showed significantly increased DNMT3A expression in the F1-generation pups, this effect was lost in the F3 generation. Moreover, while the expression of chromatin condensing protein protamine 1 was impaired and occurred in diplotene spermatocytes of the HFB and HFB + BPA group [15,33], in F1-generation males (under the T + E2 model), the expression pattern of protamine 1 was normal in all groups for F3 males (expressed in spermatozoa; data not shown). This might be the reason early spermatogenesis was affected in HFB and HFB + BPA groups in the F1 generation, but only later stages (round spermatids) were affected in F3 groups. Overall, our results support earlier findings of transgenerational effects of F0 BPA exposure on reduced fertility in the F3 generation of male rats [64] and female mice [65], on differences in social behavior [30,66] in mice, and on a mixture of phenotypes including obesity, infertility, and kidney disease in F3 rats in utero exposed to a combination of BPA and two phthalates [67].

Using T + E2 models that mimic aging, combined HFB + BPA exposure in the F1 generation in utero resulted in transgenerational effects in the testes of the F3-generation offspring, as evidenced by the following: (1) impaired spermatogenesis, (2) increased acrosomal CYP19A1 localization in round spermatids, and (3) increased nuclear ERbeta localization. However, while the T + E2 model showed significantly impaired spermatogenesis in the F3-generation offspring, the naturally aged F3 offspring in the BPA, HFB, and HFB + BPA groups showed a trend toward impaired spermatogenesis, but this lost significance compared to the AIN diet. This study gives hope that while the deleterious effects of a diet rich in butterfat and BPA can be inherited across generations, its effects are weakened in the F3 generation. Furthermore, due to an inherent limitation in our study (small animal size), additional studies are necessary to support our results.

**Figure 9:**
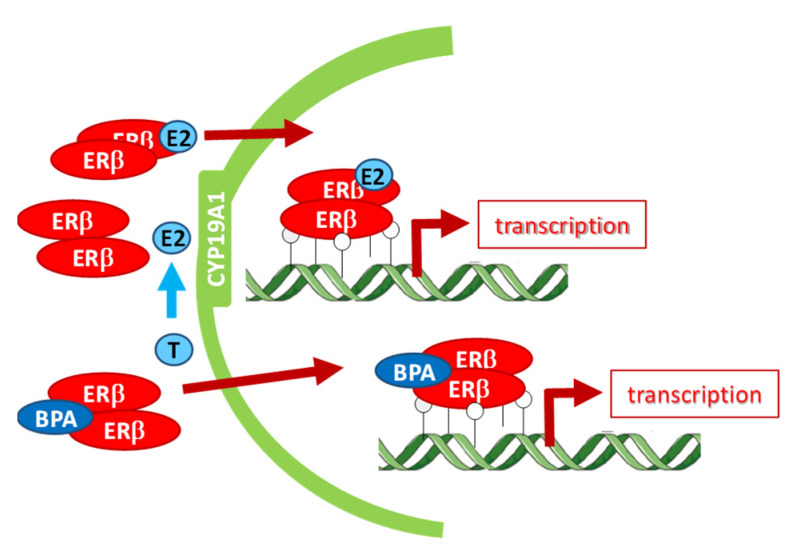
Schematic explaining mechanism of action of ERbeta and CYP19A1 interaction at the acrosome. T, testosterone; E2, estradiol. CYP19A1catalyzes conversion of T to E2. E2 binds to ERbeta (ERβ). ERbeta-E2 enters the nucleus and regulates expression of genes involved in cell survival and apoptosis.

Evidence from transgenic overexpression and knockout studies suggest the involvement of ERbeta and CYP19A1 in normal spermatogenesis within the testes [68,69,70,71,72,73]. We found that CYP19A1 was strongly localized to the acrosome in BPA, HFB, and HFB + BPA groups and that acrosomal ERbeta localization correlated with nuclear ERbeta localization. We thus speculated that increased CYP19A1 localization to the acrosome of round spermatids acts as a conduit to direct ESR2/ERbeta into the cell nucleus, where it functions to downregulate genes involved in spermiogenesis (Figure 9). In support of this, it was previously suggested that the spermiation failure and increased spermatocyte cell death via ERbeta was due to increased oxidative stress, decreased expression of genes involved in actin remodeling, and decreased transcripts of anti-apoptotic genes [61]. Also, overexpression of ERbeta has been shown to result in germ cell cycle arrest, cell death, and infertility [74]. Estrogen has been found to regulate, through the ERbeta pathway, genes controlling round spermatid differentiation in rats [75]. Moreover, overexpression of CYP19A1 in male mice was shown to increase estrogen production and cause infertility in adulthood [76]. Hence, dysregulation of ERbeta and CYP19A1 expression has been associated with increased probability of infertility.

BPA has been found to cause alterations in zebrafish reproduction by decreasing global DNA methylation [77] and causing dysregulation of epigenetic remodeling enzymes [78]. Other labs found that BPA interfered with the reproductive process in zebrafish females by deregulating histone modification and DNMT gene expression [79]. Similarly, in male zebrafish, histone acetylation was found to be enhanced in spermatozoa and embryos from males exposed to BPA treatment [60,78]. Chronic exposure of zebrafish to BPA for two generations was found to affect sperm quantity and quality in F1- and F2-generation adults [80]. In mice, decreased CpG methylation of the IAP sequence upstream of the Agouty gene was observed, implicating possible transgenerational effects of BPA [81]. 

Studies from our laboratory found that in the rat prostate gland [58], neonatal exposure to estradiol/BPA alters the transcriptional program of factors involved in DNA methylation (DNMT3A/B and MBD2/4) and hypomethylation of the promoter of nucleosome binding protein-1 (NSBP1), an early and permanent epigenetic mark of neonatal exposure to estradiol/bisphenol A that persists throughout life. Additionally, maternal high-fat diets in mice have been found to be associated with altered gene expression, chromatin marks, and DNA methylation changes [82,83,84] in male and female offspring. However, in our present study, we did not observe any consistent changes in transcripts for the DNA methyl transferases, or for the methyl-CpG-binding domain proteins *MBD1, MBD2*, or *MBD4* between the various groups in the F1- and F3-generation offspring. While we have not analyzed global DNA methylation changes within the different testis cell types, our current data suggest that changes in DNA methylation may not be the principal epigenetic regulator at this low concentration of BPA (25 µg/kg bw). Here, we found that expression levels of *MBD3* decreased in HFB and HFB + BPA groups compared to AIN in both the F1 and F3 generation. MBD3 is an integral component of the NuRD, a multisubunit complex involved in nucleosome remodeling and histone deacetylase activity. While the target genes for MBD3 have not been identified in germ cells, depletion of MBD3 was found to induce arrest at the G2/M transition and result in defective mitosis in cancer cells. Chromatin immunoprecipitation analysis revealed that the transcription of genes involved in cell cycle regulation and anti-apoptosis (CylinB1, Plk1, and Survivin/BIRC5) is modulated by MBD3 [85]. MBD3-NuRD is also involved in the transcriptional repression of Netrin-1 [86] and PYCARD [87], both of which are involved in apoptosis. The mechanism through which the decrease in MBD3 level is involved in increased CYP19A1/ERbeta expression in germ cells, and/or localization of Cyp19A1 to acrosomes, is currently under investigation.

In summary, exposure of pregnant rats to HFB, BPA, and HFB + BPA resulted in the development of adult-onset disease in the testes of T + E2-treated and middle-aged F1 male offspring. This phenotype developed transgenerationally in males of the F3 generation, with significance restricted to the T + E2-treated HFB + BPA group. However, the epigenetic mechanism responsible for the maintenance of the disease phenotype across generations that “switched on” in the testes with age remains to be determined.

## Figures and Tables

**Table 1:** Antibodies used in this study.

Peptide/Protein Target	Antigen Sequence	Name of Antibody	Manufacturer, Catalog Number	Species, Monoclonal or Polyclonal	Dilution
CYP19A1	209-503	H-300	Santa Cruz Biotechnology, sc-30086	Rabbit polyclonal	1:50
ESR2/ERbeta	17-mer, close to C-terminus	anti-ESR2	BioGeneX, AR385	Rabbit polyclonal	1:100

**Table 2:** Primers used for real-time PCR.

Primer Name		Primer Sequence	Amplicon Size (bps)	Annealing Temperature
*rDNMT1*	Forward:	5′-GAGGTGGGCGACTGCGTCTC-3′	214	60
	Reverse:	5′-TGTGGATGTAGGAAAGTTGCA-3′		
*rDNMT3a*	Forward:	5′-CAGAATAGCCAAGTTCAGCAAAGTGA-3′	68	58
	Reverse:	5′-CTTTGCCCTGCTTTATGGAG-3′		
*rDNMT3b*	Forward:	5′-GTTAAAGAAAGTACAGACAATAACCAC-3′	220	57
	Reverse:	5′-TCTGATGACTGGCACACTCC-3′		
*rDNMT3l*	Forward:	5’-AATGGCCGAAATCAGCCCCA-3’	139	60
	Reverse:	5’-CGCTGGTTCACGTTGACTTC-3’		
*rMeCP2*	Forward:	5′-GTCGCTCTGCTGGAAAGTAT-3′	189	57
	Reverse:	5′-TGGGCTTCTTAGGTGGTTTC-3′		
*rMBD1*	Forward:	5′-CAGCAGTCACAACCTTCCTG-3′	182	58
	Reverse:	5′-GGTGCCAATCCCTCCTATCT-3′		
*rMBD2*	Forward:	5′-GTCGGCCCAGGTAGTAATGAT-3′	195	60
	Reverse:	5′-GACTCGCTCTTCCTGTTTCCT-3′		
*rMBD3*	Forward:	5′-CTGAACACTGCACTGCCTGTA-3′	145	58
	Reverse:	5′-GTTTCTTCTCCCAGAAAAGCTG-3′		
*rMBD4*	Forward:	5′-CCTACCGGATCTTTTGTGTCA-3′	90	58
	Reverse:	5′-GATTTTCCCAAAGCCAGTCAT-3′		
*rMBP3l1*	Forward:	5’-GCTGGTTGGAGACTGGCAAT-3’	96	60
	Reverse:	5’-TTGCCCATCTGACTCCGTTC-3’		
*rRpl19*	Forward:	5′-GCATATGGGCATAGGGAAGA-3′	197	58
	Reverse:	5′-CCATGAGAATCCGCTTGTTT-3′		

**Table 3:** Presence of spermatozoa in ≥14% of STs (T + E2) in testes of F3-generation SD rats.

	Number of Animals	% Animals (Normal)	Odds Ratio	*p*-Value
AIN	10/11	91%		
BPA	10/14	71%	4	0.340
HFB	9/14	64%	5.6	0.180
HFB + BPA	6/15	40%	15	0.014 *

*p*-Value from Fisher’s exact test (two-tailed) compared with AIN diet. * *p* < 0.05.

**Table 4:** Presence of spermatozoa in >20% of STs in testes of F3-generation SD rats (aging study).

	Number of Animals	% Animals (Normal)	Odds Ratio	*p*-Value
AIN	6/7	85.7%		
BPA25	4/5	80.0%	1.5	1
HFB	4/5	80.0%	1.5	1
HFB + BPA	3/6	50.0%	6.0	0.265

*p*-Value from Fisher’s exact test compared with AIN diet.

## Data Availability

Not applicable.
